# Analysis of stakeholder networks for breastfeeding policies and programs in Ghana

**DOI:** 10.1186/s13006-020-00311-x

**Published:** 2020-08-24

**Authors:** Richmond Aryeetey, Kassandra Harding, Amber Hromi-Fiedler, Rafael Pérez-Escamilla

**Affiliations:** 1 University of Ghana School of Public Health, Legon, Accra Ghana; 2 Department of Health Science & Human Ecology, California State University, San Bernardino, California USA; 3 Yale School of Public Health, New Haven, CT USA

**Keywords:** Actor, Influence, Policy, Network, Mapping, Breastfeeding, Ghana

## Abstract

**Background:**

Suboptimal breastfeeding practices are driven by multiple factors. Thus, a multi-sectoral approach is necessary to design and implement appropriate policies and programs that protect, promote, and support breastfeeding.

**Methods:**

This study used Net-Map, an interactive social network interviewing and mapping technique, to: a) identify key institutional actors involved in breastfeeding policy/programs in Ghana, b) identify and describe links between actors (i.e., command, dissemination, funding, and technical assistance (TA)), and c) document actors influence to initiate or modify breastfeeding policy/programs. Ten experts were purposively selected from relevant institutions and were individually interviewed. Interview data was analysed using social networking mapping software, *Gephi* (version 0.9.2).

**Results:**

Forty-six unique actors were identified across six actor categories (government, United Nations agencies, civil society, academia, media, others), with one-third being from government agencies. Dissemination and TA links accounted for two-thirds of the identified links between actors (85/261 links for dissemination; 85/261 for TA). Command links were mainly limited to government agencies, while other link types were observed across all actor groups. Ghana Health Service (GHS) had the greatest in-degree centrality for TA and funding links, primarily from United Nations Children’s Fund (UNICEF) and development partners. The World Health Organization, UNICEF, Ministry of Health, and GHS had the highest weighted average relative influence scores.

**Conclusions:**

Although diverse actors are involved in breastfeeding policy and programming in Ghana, GHS plays a central role. United Nations and donor agencies are crucial supporters of GHS providing breastfeeding technical and financial assistance in Ghana.

## Background

Policies and programs that protect, promote, and support breastfeeding represent an important, cost-effective pathway to improve human health and development outcomes [1, 2]. Despite the well-documented benefits of breastfeeding to people, societies, and the planet [1], optimal breastfeeding practices are often challenged by multiple adverse socio-cultural barriers as well as sub-optimal policy design, and program implementation. Current evidence demonstrates that most women are not surrounded by friendly and supportive environments for breastfeeding, due in part to lack of social support, inadequate funding, suboptimal monitoring and enforcement of legislation, and limited institutional capacity to support and protect optimal breastfeeding practices [3]. The barriers to optimal breastfeeding practices are multi-factorial and complex. Thus, efforts to scale up national breastfeeding programs require multi-faceted strategies that are well-coordinated across multiple institutional actors at the country level [4–6]. Yet, there is a dearth of empirical evidence on how diverse decision makers interact, dynamically, while implementing multi-faceted breastfeeding policies and programs within lower income country settings [7].

Although Ghana has experienced substantial improvements in breastfeeding practices in the past, the country’s capacity to sustain these gains has been limited. More than a decade ago, Ghana was considered one of the few breastfeeding success stories in Africa [8]. Success in breastfeeding initiation and exclusive breastfeeding in Ghana were attributed to behaviour change communication interventions that were implemented as part of an integrated program that also included intersectoral partnerships, capacity building, and community engagement [9]. The adoption of national policies also contributed to the improvement of breastfeeding outcomes in Ghana, beginning in 1992, with the adoption of the International Code of Marketing of Breastmilk Substitutes [9]. Between 1993 and 2003, Ghana adopted additional supportive breastfeeding policies (i.e. Baby-Friendly Hospital Initiative, National Breastfeeding Policy, Breastfeeding Promotion Regulation, maternity protection provisions in the National Labour Law) [9], which translated into stronger breastfeeding outcomes over the next decade. The national Labour law [10] incorporates maternity protection provisions of the International Labour Organization (ILO).

Exclusive breastfeeding rates in Ghana climbed to an apex in 2008, reaching 63%. However, national surveys since then have documented a steady decline in exclusive breastfeeding rates to 52% in 2014 and then 43% in 2017. The prevalence of timely initiation of breastfeeding has also remained low and is currently at 56% [11, 12].

These downward trends prompted an examination of the country’s commitment to address the needs to improve their national breastfeeding policies and programs. In 2016, the Becoming Breastfeeding Friendly (BBF) process was implemented in Ghana resulting in the identification of several gaps in the national breastfeeding environment that limited effective scale-up of breastfeeding policies and programs [13]. A BBF committee, which evaluated the evidence on the breastfeeding programming situation, proposed recommendations to fill such gaps [13]. The BBF committee comprised of key breastfeeding implementation and technical stakeholders in Ghana across academia (e.g. University of Ghana), civil society (e.g. Ghana Infant Nutrition Action Network), government (e.g., Ghana Health Service; Ministry of Gender, Children and Social Protection), and international organizations (e.g. World Food Program, UNICEF, WHO). To effectively and efficiently implement the policy and program recommendations proposed by the BBF committee, it was realized that an understanding of the roles and links between stakeholders in the breastfeeding landscape was needed.

Stakeholder analysis, power mapping, and social network analysis are tools that have been used previously to identify stakeholders and their influence on policies across diverse fields [14–16]. The International Food Policy Research Institute developed the Net-Map methodology which combines these three analytical methods [14]. Net-Map has been successfully applied to examine stakeholder characteristics regarding infant and young child feeding policies and programs in Asia [17, 18].

Given the recent evidence-informed BBF recommendations for strengthening the breastfeeding enabling environment in Ghana [13], the application of Net-Map can help understand the landscape of stakeholders involved in the breastfeeding arena and inform actions to support the scaling up of effective national breastfeeding policies and programs in Ghana. Thus, the aim of this analysis was to: a) map the landscape of stakeholders (i.e. actors) involved in breastfeeding programs and policy in Ghana; b) document relationships between stakeholders; c) understand their influence on breastfeeding programs and policy.

## Methods

### Study setting

Ghana is a West African country with relatively high rates of acute and chronic malnutrition among children < 5 years of age [11]. Exclusive breastfeeding rates are low at 43%, with the median duration of exclusive breastfeeding being 2.5 months [12]. While the median duration of predominant breastfeeding is approximately 4 months, the median duration of any breastfeeding extends to almost 20 months [12]. Predominant breastfeeding occurs when the infant’s predominant source of nourishment is breast milk (including milk expressed or from a wet nurse) as the predominant source of nourishment; but infant may also receive liquids (water and water-based drinks, fruit juice), as well as ritual fluids and ORS, drops or syrups (vitamins, minerals and medicines) [19].

### Study design and approach

Ten experts from government and non-government institutions at the national level participated in Net-Map interviews between December 2017 and January 2018. Respondents were selected, purposively, from an initial list of 31 experts that had attended BBF meetings, were on the BBF committee, or who were identified by the in-country BBF director as being instrumental in strengthening the breastfeeding environment in Ghana [13]. The 31 experts were ranked by the country BBF director according to the level of influence they have within their organizations for decision making regarding breastfeeding. The top 10 most influential experts were invited to participate in the interviews. All interviews were carried out in-person in Accra, the capital of Ghana, and most were held at the participant’s (i.e., expert’s) office within a confidential area. Interview lasted, on average, almost 2 h (minimum was 1.5 h and maximum was 2 h).

Each interview consisted of three steps: 1) identifying the stakeholders (i.e. actors) involved in providing breastfeeding protection, promotion, and support services in Ghana, 2) generating a directed map of actor networks via pre-defined relationships, or link types (i.e. command, dissemination, funding, and technical assistance), and 3) assigning an estimate of influence to each actor, relative to other actors, within the breastfeeding landscape. Three maps were generated for each interview documenting actors, links, and influence, respectively. Interviews were facilitated to avoid repetition from previous paragraph were facilitated by a lead interviewer with the support of at least one research assistant. Data collected at each interview included photographs of the maps, hand written notes, and an audio recording.

At the start of the interview, each participant was asked to first identify, *who* was involved in breastfeeding decision-making in Ghana (at all levels including the national and sub-national levels). The names of the identified actors, principally institutions, were written boldly on color-coded ‘stick-on’ paper that specified the actor’s group identified by the participant: government (pink), United Nations agencies (blue), non-government organizations (yellow), academic/research (green), and others (orange). The stick-on paper for each actor was then placed on a large sheet of paper to show all the actors involved within the breastfeeding environment.

Once all actors had been identified, participants were then asked *how* the actors were linked to each other. Participants were instructed to identify and label links as command, dissemination, funding, or technical assistance (Table 1). In addition to the pre-defined link types, advocacy emerged as a posteriori link type. Links were displayed as arrows between two actors, indicating direction of link. For example, a command link going from actor A to actor B indicated that actor A provides command/instruction to actor B.

**Table 1:** Description of links assessed in the Net Map interviews in Ghana

Link type	Operational definition
Funding (F)	Any two or more actors/institutions are linked by giving or receiving money or financial incentives (for example, one institution funds projects of another). More specifically, a funding link going from actor A to actor B indicates that actor A provides funding to actor B.
Command (C)	Any two or more actors/institutions are linked by giving or receiving commands/directives (for example, one institution tells the other that it must implement an activity). More specifically, a command link going from actor A to actor B indicates that actor A provides command/instruction to actor B.
Technical Assistance (TA)	Any two or more actors/institutions are linked by giving or receiving technical assistance (for example, one institution offering advice/skilled-based training to another). More specifically, a technical assistance link going from actor A to actor B indicates that actor A provides technical assistance to actor B.
Dissemination (D)	Any two or more actors/institutions are linked by dissemination of information (for example, one or both institutions spread information that one or both have developed). More specifically, a dissemination link going from actor A to actor B indicates that actor A disseminates information to actor B.
Advocacy (A)	Any two or more Actors/institutions are linked by advocacy actions (for example one institution influences decisions of the other through lobbying, research, public education etc). More specifically, an advocacy link going from actor A to actor B indicates that actor A advocates to actor B.
Network density	Proportion of the total links identified in a network out of the total possible links of a network [20].
Network distance	Average of the shortest directed lengths between two actors. Measures of centrality identified the prominent actors within a network [20].
In-degree centrality	The number of links directed at an actor, representing the received input from a particular network [20]. For example, in the network describing the links between actors representing commands, in-degree centrality quantified the number of other actors in the network from which commands, or directions, are received by a particular actor.
Out-degree centrality	The number of links from one actor directed to other actors in the network, representing the input provided to a particular network [20]. In the example of command, out-degree centrality quantified the number of command links of a particular actor provided to other actors in the network.
Betweenness centrality	The number of times an actor lies on the shortest path between two other actors within a network, representing the control an actor has over the flow of inputs across a network [20].

Finally, participants were asked *how much* influence each actor had with respect to developing or implementing breastfeeding policies and programs in Ghana. Participants were asked to place checker pieces in a tower on each actor on the map as a representation of the relative influence of each actor in the network. The actor with the largest number of checkers was considered as having the greatest relative influence. Ethical approval for this study was obtained from the Ethics Review Committee of the Ghana Health Service (GHS-ERC 14/06/17).

#### Data cleaning and analysis

Data from the photographed maps of the actors, links, and influence towers from each interview were entered into an Excel file. Influence towers were standardized within each interview, on a scale of 0 to 1. Actor data was compared across participant maps and recoded to ensure a consistent categorization of actor groups and actor designation. Recoding of actor groups was based on the most cited actor group, across participant maps. Recoding of some link types was required, as different link types had emerged in interviews that were not based on the pre-coded link types (e.g. advocacy).

Data of actor characteristics across the 10 interviews were merged and the number of actor citations were reported from the combined data. An average relative influence (RI) across interviews was then calculated (i.e. sum of RI across interviews /10) for each actor and weighted by number of citations (RI-W). RI-W was used as the measure of influence for each actor. Data on links between actors across the 10 interviews were appended in one dataset. The final aggregated data from the 10 interviews were imported into Gephi (version 0.9.2) for analysis.

Social network maps were configured using the Yifan Hu algorithm [21], and actor networks were mapped for each link type (i.e. command, dissemination, funding, technical assistance, and advocacy). Actor size in the networks represented the actor’s RI-W, with larger nodes (i.e. actors) representing higher RI-W. The actors were also color-coded by actor groups.

Social network analysis measures of cohesion (i.e. network density and distance) and measures of centrality (i.e. in-degree, out-degree, and betweenness) were used to evaluate the networks. Measures of cohesion provide a broader picture of the network position, while measures of centrality offer insight into the relationships (via link type) between actors. Distribution of actor groups (eg, government, non-governmental organizations, development partners, etc.) was also reported and evaluated across networks.

## Results

### Characteristics of actors

**Fig. 1:**
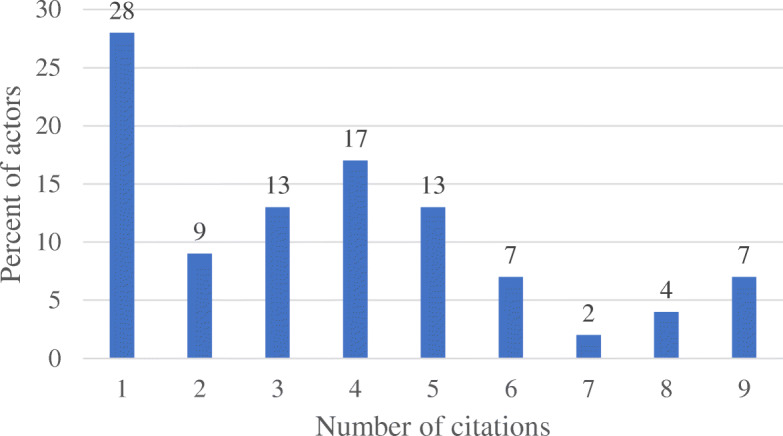
Frequency of actor citations across interviews in Ghana^1^. ^1^ This figure describes the percent of the actors that were cited in all interviews and how many times they were cited (i.e. between 1 and 9 times). For example, 28% of the actors cited in the interviews were cited only once by interview participants

The ten interview respondents represented government institutions (*n* = 4), United Nations agencies (*n* = 3), international non-governmental organizations (*n* = 2), and an implementing agency of a bilateral donor agency in Ghana (*n* = 1). All respondents were females and working as program officers in their respective institutions. Four out of the 10 respondents had training in nutrition and all had roles linked with child health and nutrition. Across the 10 interviews, 46 unique actors who engaged with breastfeeding promotion, protection, and support, were identified (additional file 1) into six actor groups including government agencies (41.3%), non-governmental/civil society organizations (26.1%) and United Nations agencies (10.9%) (Table 2). One-third (36.9%) of the actors were institutions which focus their activities on health policy, services and regulation. Apart from the First Lady, all actors were institutions. Frequency of actors mentioned across the 10 interview respondents ranged from 1 to 9 (Fig. 1). One-third of the actors (33.0%) were mentioned in at least 5 of the 10 interviews. Ghana Health Service (GHS), Ministry of Health (MOH), and Parliament, were most frequently mentioned actors; they were mentioned in nine interviews. About 28% of actors were mentioned in only one interview.

**Table 2:** Summary of Actor Groups in Ghana (*N* = 46)

	Frequency of unique actors cited	% of unique actors cited
Government	19	41.3
NGO/CSO	12	26.1
United Nations Agencies	5	10.9
Academia^1^	1	2.2
Media^2^	1	2.2
Other^3^	8	17.4

Abbreviations: Non-Government organization/civil society organization (NGO/CSO)

^1^Academia represents multiple higher education institutions

^2^Media represents multiple media organizations

^3^Includes private-for-profit institutions, organized labour, nutrition champion (First Lady), donor agencies, implementing agencies of donor agencies

### Actor influence

**Fig. 2:**
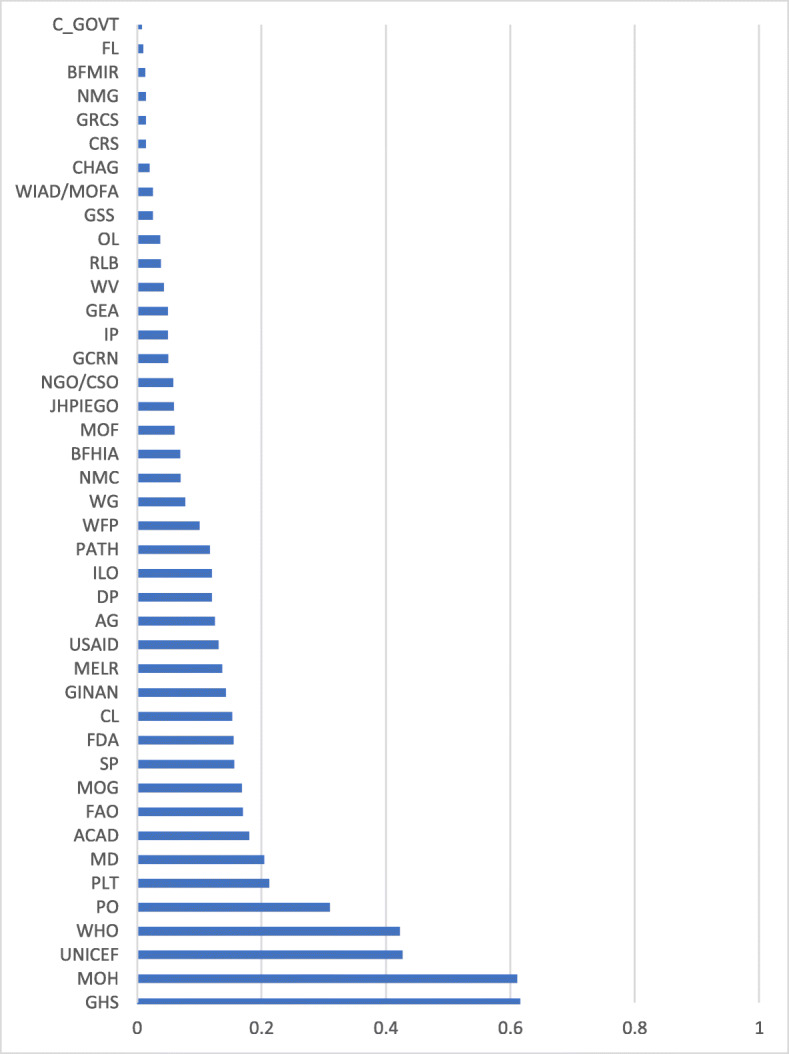
Weighted average relative height of influence towers for all actors in Ghana, ranked lowest to highest (those identified with 0 influence have been excluded from this map, n = 4 excluded). Abbreviations: Academia (ACAD); Attorney General (AG); Baby Food Manufacturers/Importers/Retailers (BFMIR); Baby-friendly Hospital Initiative Authority (BFHIA) Catholic Relief Services (CRS); Central Government (C_GOVT); Christian Health Association of Ghana (CHAG); Community Leaders (CL); Development Partners (DP); First Lady (FL); Food and Agricultural Organization (FAO); Food and Drug Administration (FDA); Ghana Community Radio Network (GCRN); Ghana Employment Association (GEA); Ghana Health Service (GHS); Ghana Infant Nutrition Action Network (GINAN); Ghana Red Cross Society (GRCS); Ghana Statistical Service (GSS); Implementing partners (IP); International Labour Organisation (ILO); John Hopkins Program for International Education in Gynecology and Obstetrics (JHPIEGO); Media (MD); Ministry of Employment and Labour Relations (MELR); Ministry of Finance (MOF); Ministry of Gender and Social Protection (MOG); Ministry of Health (MOH); Non-governmental Organization/Civil Society Organization (NGO/CSO); Nurses and Midwifery Council (NMC); Nurses and Midwives Groups (NMG); Organized Labour (OL); Parliament (PLT); Program for Appropriate Technology in Health (PATH); Public Opinion (PO); Regulatory Bodies (RLB); Service providers (SP); United State Agency for International Development (USAID); Women in Agricultural Development/Ministry of Food and Agriculture (WIAD/MOFA); Women’s Groups (WG); World Health Organization (WHO); World Vision (WV); United Nations Children’s Fund (UNICEF)

RI-W among actors ranged from 0 to 0.62 (Fig. 2). GHS (0.62) and MOH (0.61) were the actors with the highest RI-W, followed by UNICEF (0.43) and WHO (0.42). Among the remaining actors, 17 received a RI-W ≥ 0.10 and < 0.40, 21 received a RI-W between > 0 and < 0.10, and 4 actors received a RI-W of zero, indicating, they have no influence over breastfeeding policy and programs, relative to the other actors.

### Actor networks

**Fig. 3:**
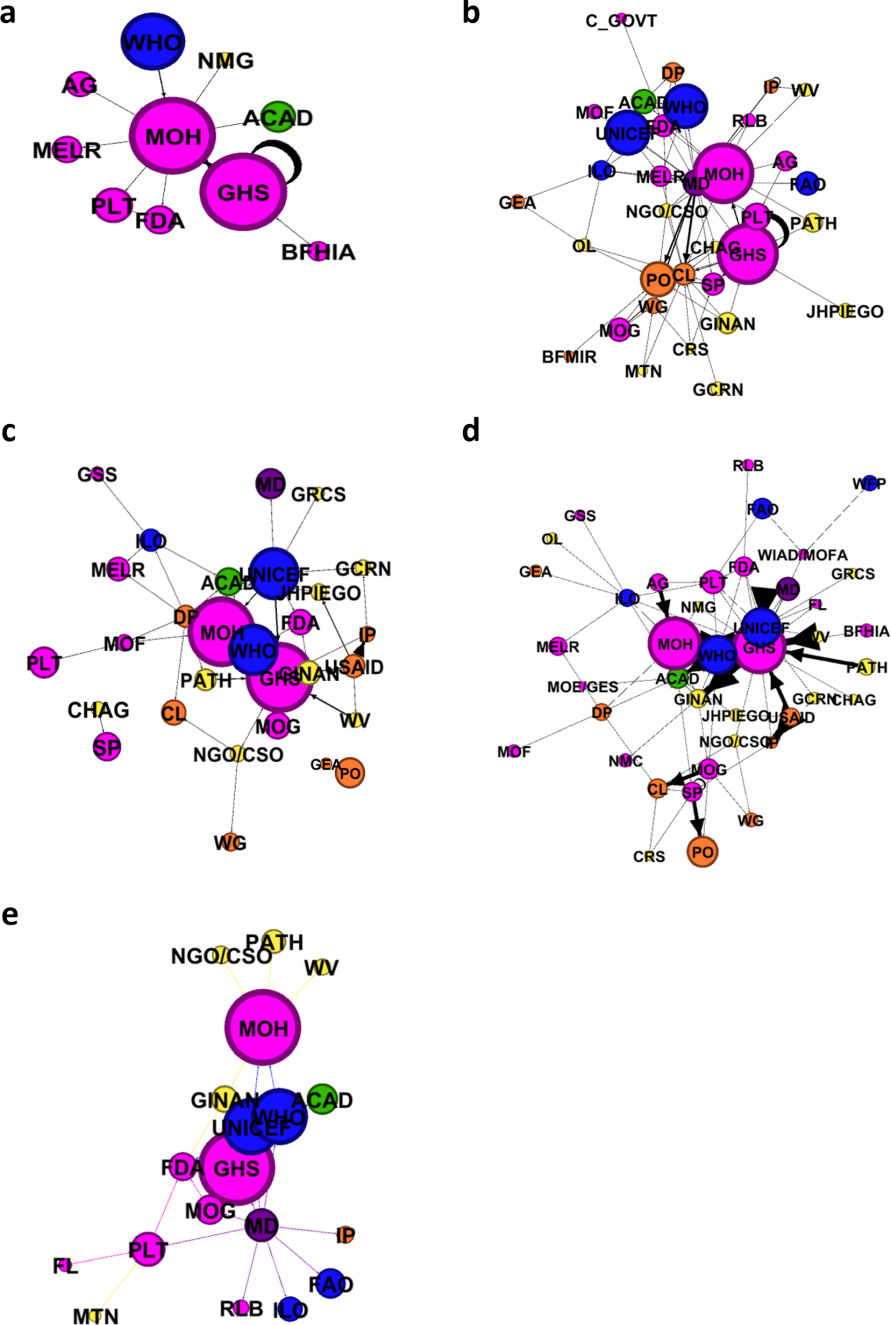
Map of Ghana’s breastfeeding stakeholders sized by weighted average reported influence, color coded by actor groups, and stratified by link time. A. Command links: 12 actors, 14 unique links; b. Dissemination links: 34 actors; 85 unique links; c. Funding links: 29 actors; 49 unique links; d. Technical Assistance links: 41 actors, 85 unique links; e. Advocacy links: 19 actors; 28 unique links. Pink = Government; Yellow = NGO; Blue = UN Agencies; Green = Academia; Purple = Media; Orange = Other Abbreviations: Academia (ACAD); Attorney General (AG); Association of Ghana Industries (AGI); Baby Food Manufacturers/Importers/Retailers (BFMIR); Baby-friendly Hospital Initiative Authority (BFHIA); CARE International Catholic Relief Services (CRS); Cabinet (CBT); Central Government (C_GOVT); Christian Health Association of Ghana (CHAG); Community Leaders (CL); Catholic Relief Services (CRS); Customs (CUS); Development Partners (DP); First Lady (FL); Food and Agricultural Organization (FAO); Food and Drug Administration (FDA); Ghana Community Radio Network (GCRN); Ghana Employment Association (GEA); Ghana Health Service (GHS); Ghana Infant Nutrition Action Network (GINAN); Ghana Red Cross Society (GRCS); Ghana Statistical Service (GSS); Hospitals (HOSP); Implementing partners (IP) International Labour Organisation (ILO); John Hopkins Program for International Education in Gynecology and Obstetrics (JHPIEGO); Media (MD); Ministry of Employment and Labour Relations (MELR); Ministry of Education/Ghana Education Service (MOE/GES); Ministry of Finance (MOF); Ministry of Gender and Social Protection (MOG); Ministry of Health (MOH); Mobile Telecommunications Network (MTN); Non-governmental Organization/Civil Society Organization (NGO/CSO); Nurses and Midwifery Council (NMC); Nurses and Midwives Groups (NMG); Organized Labour (OL); Parliament (PLT); Program for Appropriate Technology in Health (PATH); Public Opinion (PO); Regulatory Bodies (RLB); Service providers (SP); United State Agency for International Development (USAID); Women in Agricultural Development/Ministry of Food and Agriculture (WIAD/MOFA); Women’s Groups (WG); World Health Organization (WHO); World Vision (WV); United Nations Children’s Fund (UNICEF)

Five networks were generated demonstrating command, dissemination, funding, technical assistance, and advocacy linkages (i.e. relationships) between actors in the breastfeeding landscape in Ghana (Fig. 3). A range of network size and density was observed. While a total of 46 actors were identified across the five networks, the number of actors within different networks ranged from 12 actors in the network for command, to 41 actors in network for technical assistance. (Table 3). Each network is described below.

**Table 3:** Summary of breastfeeding stakeholder networks in Ghana, by linkages

Network	Number of links	Number of actors	Greatest Betweenness Centrality	Greatest Out-Degree Centrality	Greatest In-Degree Centrality
Advocacy	28	19	GINAN (3.0)	MD (9)	MOH (6)
Command	14	12	MOH (19.0)	MOH (7)	GHS (3)
Dissemination	85	34	MD (232.9)	MD (16)	CL (13)
Funding	49	29	GHS (86.5)	DP (9)	GHS (10)
Technical Assistance	85	41	GHS (298.1)	UNICEF (10)	GHS (13)

Abbreviations: Community leaders (CL); Development partners (DP); Ghana Health Service (GHS); Ghana Infant Nutrition Action Network (GINAN); Ministry of Health (MOH); Media (MD); United Nations Children’s Fund (UNICEF)

### Command

A total of 12 actors were connected by 14 unique command links (Fig. 3a). The majority of the actors in this network were from the government sector (*n* = 7). The network density was 0.20, thus 20% of possible links across the network had been achieved. The average distance between any two actors was 1.81. The MOH was a central actor in the command network, with the greatest betweenness centrality, suggesting an important role in connecting other actors in the network. The MOH also provided input, or command, to the largest number of other actors in this network (out-degree centrality: *n* = 7 links). Most of the actors that MOH provided command to (*n* = 5) were other government actors. GHS received command from the largest number of other actors (in-degree centrality: *n* = 3 links), all of whom were government actors. Two actors and community leaders were identified as providing command to each other, but not connected to the other actors in the network.

### Dissemination

The dissemination network included 85 unique links that connected 34 actors (Fig. 3b). These links encompassed all the six actor groups. The network density was 0.14 (14% of possible links across the network had been achieved) and the average distance between any two actors was 2.36. The media had the highest betweenness centrality, serving as an important connector across actors, and also provided dissemination to the largest number of other actors in the network (out-degree centrality: *n* = 16 links). The actors to whom the media provided dissemination included those within government, United Nations (UN), non-governmental organizations (NGOs), and other actor groups. Among the actors, community leaders (CL) received dissemination from the greatest number of other actors (in-degree centrality: *n* = 13 links), who represented a variety of actor groups, including the media, NGOs, and government actors.

### Funding

The funding network comprised of 29 actors connected by 49 unique links (Fig. 3c). All six actor groups were represented in the Funding network. The density of the network was 0.11 and the average distance between any two actors was 2.27. There were two pairs of actors who were connected to each other, yet not connected to the larger network: Ghana Employer’s Association provided funding to raise public opinion about breastfeeding, and Christian Health Association of Ghana provided funding to service providers. GHS played a central role in connecting various actors. GHS received funding from the greatest number of actors in the network (in-degree centrality: *n* = 10 links) and was, in turn a source of funding to NGO/Civil society organizations (NGO/CSO), MOH, and Ghana Infant Nutrition Action Network (GINAN). Development partners (DP) were represented as a single actor, although comprising of multiple donors, provided funding to the highest number of actors (out-degree centrality: *n* = 9 links), including actors from government, UN, academic, NGO, and other actor groups.

### Technical assistance

The technical assistance network included 85 unique links between 41 actors, who represented all actor groups (Fig. 3d). The network density was 0.09, indicating that less than 10% of possible connections in the network existed. The average distance between any two actors was 2.67. GHS received technical assistance from the greatest number of actors (in-degree centrality: *n* = 13 links) across including USAID, Academia, WHO, and UNICEF. In turn, GHS provided technical assistance to 9 other actors. UNICEF provided technical assistance to the greatest number of actors in the network (out-degree centrality *n* = 10 links). In addition to GHS, UNICEF provided technical assistance to government, academic, NGO, UN, and media (MO) actors.

### Advocacy

The advocacy network consisted of 19 actors connected by 28 unique links (Fig. 3e). Advocacy links were reported among all actor groups but were mostly between government, NGO/CSO, and UN actors. The network density was 0.16 and the average distance between two actors was 1.26. MOH received advocacy from the largest number of actors in the network (in-degree centrality: *n* = 6 links), while the media advocated to the largest number of actors (out-degree centrality: *n* = 9 links). The media’s advocacy efforts were primarily focused on UN actors (*n* = 4), government actors (*n* = 4) and implementing partners (*n* = 1).

## Discussion

Through these analyses, the size, composition, and network of the national breastfeeding stakeholder landscape was identified across links of command, dissemination, funding, technical assistance, and advocacy. The density of the networks ranged from 0.09 (technical assistance) to 0.20 (command), indicating that only as much as 20% of possible links in these networks exist. Furthermore, in the networks for command and funding, there were two-actors isolated from the larger network of actor within these two areas. While it would be inefficient to have a network density of 1.00 as certainly every actor need not to be connected to every other actor, a lower network density can place greater importance on actors who can connect other pairs of actors (i.e. actors with high betweenness centrality). Thus, strategically generating new and meaningful links between actors can help strengthen the breastfeeding environment by increasing the efficient flow of information and resources across relevant actors.

Unlike the landscape observed in India, where substantial proportion of actors had high relative influence [22], few actors in the current analysis were identified through the network and power mapping as influential actors. Two government partners, GHS and MOH, had the highest RI-W. These two actors were central in all of the networks analysed, as both a source and target for command as well as a target for funding and technical assistance with regards to breastfeeding. Furthermore, these actors were identified as important gatekeepers, or connectors, in many of the networks including command, funding and technical assistance (i.e. higher betweenness centrality). UNICEF and WHO, two UN agency actors, also received high RI-W across interviews and were present in all the networks mapped, with the exception that UNICEF was not cited in the command network. However, these actors were not as central as GHS and MOH in the social network analysis. The media ranked within the top 15% of RI-W (7th out of 49) and was an important connector in the dissemination as well as advocacy networks. Interestingly, development partners (represented as a singular actor) were not identified as being influential, yet they provided funding for breastfeeding to the largest number of actors. These results suggest that there are various actors that are important within different networks, but that GHS and MOH are the two critical institutions that are involved in the full landscape of breastfeeding programs and policy in Ghana.

While there were some similarities in the central actors across networks (i.e. GHS and MOH), as indicated above, most of the networks included a diverse spread of actor groups. This diversity of actors has been previously demonstrated by social network mapping of actors working in infant and young child feeding elsewhere [16, 22, 23]. In India, network analysis at both national and subnational levels reported diverse actors across both government and non-government agencies, and also spanned multiple sectors including, social protection, health and nutrition, research, health, planning, population sciences, media and trade unions [22]. Similar findings were observed in Sri Lanka [15]. This diversity can be valuable in the implementation of breastfeeding policies and programs because it allows for a multidisciplinary approach that relies on diverse partners and approaches.

Findings from the BBF toolbox previously identified suboptimal coordination of stakeholders working in the breastfeeding landscape in Ghana at the national level [13]. While this is not the first time that a focus on stakeholders has been identified as an important component of breastfeeding policy and programming in Ghana [24], the current study is the first to systematically assess how stakeholders interact with and influence each other within Ghana’s breastfeeding friendly environment. The Linkages Project implemented between 1997 and 2004 in Northern Ghana identified and built capacity of multiple actors, resulting in improved breastfeeding outcomes [24]. However, an assessment of barriers that limit adequate participation of actors is warranted as a next step to ensure that all relevant actors are sufficiently engaged with and influencing breastfeeding policy and programming in Ghana. The result of such an assessment can inform how actions are coordinated across all partners in order to optimize benefits from most actors involved in breastfeeding policy and programming.

Findings indicated that GHS is one of the most important actors in the breastfeeding landscape with respect to receiving technical assistance, command, and funding in Ghana, as indicated by high in-degree centrality and holding the highest relative influence among actors. MOH held the second highest relative influence among actors and was similarly central in specific networks including being a popular target of breastfeeding advocacy, and the greatest source of commend to other actors. Furthermore, both GHS and MOH served as connectors in networks, bridging links across various actors in the networks. This is consistent with the role that leading government health agencies are expected to play [16]. In the current landscape, a well-functioning GHS and MOH is critical for optimal breastfeeding promotion, protection and support in Ghana. It is also apparent that GHS, in particular, is dependent on other agencies for funding, direction (i.e. commands), and technical assistance, which is “forwarded” (i.e. provided) to other institutions and organisations, as demonstrated by its high in-degree and betweenness centrality of GHS. Thus, any situation that threatens access to these supporting resources to GHS may adversely affect breastfeeding program implementation at the national and subnational levels. Policies and strategies that strengthen the capacity of GHS are thus warranted.

Study findings provide empirical evidence of how stakeholders are currently connected with each other with regards to breastfeeding policy and programming in Ghana. The clarity of relationships provided by this study can also inform future research, particularly, regarding barriers of stakeholder engagement. The findings also complement those of from the BBF committee, which together provide evidence to inform breastfeeding decision-making in Ghana [13]. Decision-makers in Ghana can use the findings to determine which actors are better positioned to address policy recommendations identified in the BBF report, as well as identify areas within these networks that could be further developed to strengthen the breastfeeding friendly enabling environment.

This study had a few limitations. The current study mapped individual experiences of actor involvement in breastfeeding in Ghana. Although Net-Map interviews of individuals have been previously reported [15] and are considered valid [14], unlike a group Net-Map approach [16, 23], it may limit the precision and validity of recall of the role of actors by the respondents. In group Net-Map exercises, participants have an opportunity to calibrate their opinions and experiences with each other and to arrive at consensus on actor roles, linkages, and influence. Yet, this can mean that some voices are not heard as strongly as others. Alternatively, individual Net-Map exercises require the analyst to calculate agreement, such as the RI-W. However, each individual is allowed an equal voice. Therefore, pros and cons of groups vs. individual Net-Map experiences should be weighed to determine the best option within each study.

Within this study, the number of respondents was smaller (*n* = 10) than the previous smallest reported Net-Map interviewee size from previous studies (*n* = 17). The systematic selection of participants to include diverse, nationally representative individuals within the breastfeeding environment in Ghana supports the strength of this data. Additionally, findings are consistent with qualitative data showing the central role UN agencies and GHS have on strengthening the breastfeeding environment in Ghana [25]. Finally, the study was limited to the national level and thus could not be applied for decision-making at sub-national levels, which will require interviews at that level.

These limitations suggest that further research is needed in this area. Future studies evaluating differences between group and individual Net-Map results have the potential for understanding the contextual pros and cons of each method. Additionally, replicating our findings using a larger sample size of interviewees, especially across various levels of political administration, can strengthen and extend this study’s methodology, results, and recommendations to sub-national and local levels.

## Conclusions

The breastfeeding stakeholder landscape in Ghana has a wide array of actors involved in policy and program implementation, with government and UN agency actors identified as having the greatest influence in breastfeeding promotion and protection. Within the government, GHS is recognized as central in the breastfeeding landscape, yet it is heavily dependent on institutional partners for receiving support and resources, such as command, funding, and technical assistance. While GHS disseminates these resources to other stakeholders, the current findings suggest a need to strengthen GHS’s capacity to coordinate support to other actors who depend on it for resources (i.e. funding and technical assistance), thereby, enhancing the role and influence of the other stakeholders to support breastfeeding policy and programming in Ghana.

## Data Availability

The datasets analyzed for the current study are available from the corresponding author on reasonable request.

## References

[1] Victora CG, Bahl R, Barros AJD, França GVA, Horton S, Krasevec J (2016). Breastfeeding in the 21st century: epidemiology, mechanisms, and lifelong effect. Lancet.

[2] Schultink W. Why nutrition and breastfeeding are crucial to sustainable development. In: UNICEF Connect. 2015. https://blogs.unicef.org/blog/why-nutrition-and-breastfeeding-are-crucial-to-sustainable-development/. Accessed 22/02/19.

[3] Rollins NC, Bhandari N, Hajeebhoy N, Horton S, Lutter CK, Martines JC (2016). Why invest, and what it will take to improve breastfeeding practices?. Lancet.

[4] Perez-Escamilla R, Curry L, Minhas D, Taylor L, Bradley E: Scaling up of breastfeeding promotion programs in low- and middle-income countries: the "breastfeeding gear" model. Adv Nutr. 2012, 3(6):790–800. 10.3945/an.112.002873 PMC3648703 23153733

[5] Perez-Escamilla R, Hromi-Fiedler AJ, Gubert MB, Doucet K, Meyers S, Dos Santos BG (2018). Becoming breastfeeding friendly index: development and application for scaling-up breastfeeding programmes globally. Matern Child Nutr.

[6] Hromi-Fiedler AJ, Dos Santos Buccini G, Gubert MB, Doucet K, Pérez-Escamilla R. Development and pretesting of "Becoming Breastfeeding Friendly": Empowering governments for global scaling up of breastfeeding programmes. Matern Child Nutr. 2019;15(1):e12659. 10.1111/mcn.12659 PMC7198937 30211973

[7] Rasheed S, Roy SK, Das S, Chowdhury SN, Iqbal M, Akter SM (2017). Policy content and stakeholder network analysis for infant and young child feeding in Bangladesh. BMC Public Health.

[8] Quinn VJ, Guyon AB, Schubert JW, Stone-Jiménez M, Hainsworth MD, Martin LH (2005). Improving breastfeeding practices on a broad scale at the community level: success stories from Africa and Latin America. J Hum Lact.

[9] Timpo OM: Programs and policies associated with improved exclusive breastfeeding rates in Ghana: 1989-2003. Maseter's Storrs, CT: University of Connecticut; 2007.

[10] Government of Ghana (GOG). Labour Act (Act 651) (2003). Accra.

[11] Ghana Statistical Service (GSS), Ghana Health Service (GHS), ICF International. Demographic and Health Survey 2014 (2015). Rockville, Maryland.

[12] Ghana Statistical Service (GSS). Multiple Indicator Cluster Survey (MICS 2017/2018): Survey Findings Report. 2018. Accra, Ghana GSS.

[13] Aryeetey R, Hromi-Fiedler A, Adu-Afarwuah S, Amoaful E, Ampah G, Gatiba M (2018). Pilot testing of the becoming breastfeeding friendly toolbox in Ghana. Int Breastfeed J.

[14] Schiffer E, Waale D. Tracing power and influence in networks: Net-map as a tool for research and strategic network planning. 2008. Washington, DC. International Food Policy Research Institute.

[15] Godakandage SSP, Senarath U, Jayawickrama HS, Siriwardena I, Wickramasinghe S, Arumapperuma P (2017). Policy and stakeholder analysis of infant and young child feeding programmes in Sri Lanka. BMC Public Health.

[16] Karn S, Devkota MD, Uddin S, Thow AM (2017). Policy content and stakeholder network analysis for infant and young child feeding in Nepal. BMC Public Health.

[17] Harris J, Frongillo EA, Nguyen PH, Kim SS, Menon P (2017). Changes in the policy environment for infant and young child feeding in Vietnam, Bangladesh, and Ethiopia, and the role of targeted advocacy. BMC Public Health.

[18] Senarath U, Dibley MJ (2010). The South Asia infant feeding research network (SAIFRN). Introduction Food Nutr Bull.

[19] World Health Organization (WHO). The World Health Organization's Infant Feeding Recommendation. 2020. WHO.

[20] De Brun A, McAuliffe E (2018). Social network analysis as a methodological approach to explore health systems: a case study exploring support among senior managers/executives in a hospital network. Int J Environ Res Public Health.

[21] Hu YF (2005). Efficient and high quality force-directed graph drawing. The Mathematica Journal.

[22] Puri S, Fernandez S, Puranik A, Anand D, Gaidhane A, Quazi Syed Z (2017). Policy content and stakeholder network analysis for infant and young child feeding in India. BMC Public Health.

[23] Mahmood H, Suleman Y, Hazir T, Akram DS, Uddin S, Dibley MJ (2017). Overview of the infant and young child feeding policy environment in Pakistan: federal, Sindh and Punjab context. BMC Public Health.

[24] LINKAGES. World Linkages: Ghana. 2002. Washington, DC. LINKAGES/AED.

[25] Carroll G, Atuobi-Yeboah A, Hromi-Fiedler A, Aryeetey R, Safon C, Perez-Escamilla R. Factors influencing the implementation of the becoming breastfeeding friendly initiative in Ghana. Matern Child Nutr. 2019;15(3):e12787. 10.1111/mcn.12787 PMC7199075 30665255

